# Overexpression of a constitutively active truncated form of *OsCDPK1* confers disease resistance by affecting *OsPR10a* expression in rice

**DOI:** 10.1038/s41598-017-18829-2

**Published:** 2018-01-10

**Authors:** Siou-Luan He, Jian-Zhi Jiang, Bo-Hong Chen, Chun-Hsiang Kuo, Shin-Lon Ho

**Affiliations:** Department of Agronomy, National Chiayi University, Chiayi, 600 Taiwan

## Abstract

The rice pathogenesis-related protein OsPR10a was scarcely expressed in *OsCDPK1*-silenced (*Ri-1*) rice, which was highly sensitive to pathogen infection. After inoculating the leaves with bacterial blight (*Xanthomonas oryzae* pv. *oryzae*; *Xoo*), we found that the expression of *OsPR10a* was up- and down-regulated in *OEtr-1* (overexpression of the constitutively active truncated form of *OsCDPK1*) and *Ri-1* rice plants, respectively. *OsPR10a* and *OsCDPK1* showed corresponding expression patterns and were up-regulated in response to the jasmonic acid, salicylic acid and *Xoo* treatments, and *OsPR1* and *OsPR4* were significantly up-regulated in *OEtr-1*. These results suggest that *OsCDPK1* may be an upstream regulator involved in rice innate immunity and conferred broad-spectrum of disease resistance. Following the *Xoo* inoculation, the *OEtr-1* and *Ri-1* seedlings showed enhanced and reduced disease resistance, respectively. The dihybrid rice *Ri-1/OsPR10a-Ox* not only bypassed the effect of *OsCDPK1* silencing on the susceptibility to *Xoo* but also showed enhanced disease resistance and, consistent with *Ri-1* phenotypes, increased plant height and grain size. Our results reveal that *OsCDPK1* plays novel key roles in the cross-talk and mediation of the balance between stress response and development and provides a clue for improving grain yield and disease resistance simultaneously in rice.

## Introduction

In prokaryotic and eukaryotic cells, calcium ions (Ca^2+^) are intracellular second messengers that enable the sensing of a variety of environmental and developmental stimuli through temporal and spatial fluctuations or elevations in cytosolic Ca^2+^ concentrations^1,2^. In plants, calcium-dependent protein kinases (CDPKs)^3–5^ have been characterized as calcium sensors. CDPKs are considered Ser/Thr protein kinases and consist of the following four functional domains: the variable N-terminal domain, catalytic kinase domain, autoinhibitory region, and calmodulin-like regulatory domain^6^. CDPK activity was regulated by Ca^2+^, and its autoinhibitory domain can interact with the kinase domain in the absence of Ca^2+^, resulting in the inhibition of kinase activity^1^. Removing both the autoinhibitory region and the Ca^2+^ binding domains produced a constitutively active form of CDPK^1,7^. The CDPKs are encoded by multigene families in plants^8,9^ and play important physiological roles in response to diverse environmental stresses and developmental processes^1,2,10–13^.

In response to an extracellular pathogen attack, the cytosolic Ca^2+^ concentration increases as an early reaction in plant innate immunity^14–16^. The CDPKs have been suggested to function as mediators of Ca^2+^ signals, possibly instructing plants to initiate the defense response via specific protein phosphorylation events^15,17,18^. For example, the virus-induced gene silencing of *NtCDPK2* and its homologue, *NtCDPK3*, delays and reduces the extent of necrotic symptoms in tobacco, indicating that NtCDPK2 plays a role in the hypersensitivity response to pathogens^12^. In *Arabidopsis*, the overexpression of the lipid-bodies and peroxisomal localized protein AtCPK1 results in the accumulation of salicylic acid (SA), which subsequently confers resistance to the pathogen *Fusarium oxysporum*, suggesting that lipid bodies have a function in plant innate immunity^19^.

In rice (*Oryza sativa*), only a few studies have investigated the role of the CDPKs in resistance to pathogen infection. For instance, overexpression of the constitutively active OsCPK10 in *Arabidopsis* and rice results in increased resistance to *Pseudomonas syringae* pv. *tomato* and the blast fungus *Magnaporthe grisea*, respectively^20^. The ectopic expression of the wheat powdery mildew resistance gene *TaCPK2-A* in japonica rice increased the expression of the pathogen resistance-related transcription factor OsWRKY45-1 and enhanced resistance to attack by bacterial blight *Xoo* ^21^. However, OsWRKY45-1 acts as a negative regulator of resistance to *Xoo* in the japonica rice ‘Nipponbare’^22^. The overexpression of *OsCPK12* enhances tolerance to salt stress by reducing reactive oxygen species production but increases sensitivity to a blast fungus challenge^23^. Rice OsCPK18 was identified as an upstream kinase responsible for the phosphorylation and activation of OsMPK5, leading to the inhibition of the expression of defense-related genes (*PR5*, *PR10*, and *chitinase*) and negative regulation of blast fungus (*Magnaporthe oryzae*) resistance^24^. In addition, overexpression of *OsCPK4* positively regulates salt and drought stress tolerance by reducing membrane lipid peroxidation^25^ and enhances resistance to *M. oryzae* infection by preventing fungal penetration^26^.

In our previous studies, we showed that *OsCDPK1* positively regulates salt and drought tolerance but negatively affects seedling growth and seed development^27^. The overexpression of *OsPR10a* confers enhanced resistance to *Xoo* attack^28^. In the current study, we reveal that *OsCDPK1* acts as a positive regulator of *OsPR10a*, indicating that OsCDPK1 is an upstream component of the defense signaling pathway. In addition, the dihybrid rice *Ri-1/OsPR10a-Ox*, which is derived from a cross between an *OsPR10a* overexpression line (*OsPR10a-Ox*) and an RNA interference knockdown of *OsCDPK1* line (*OsCDPK1-Ri*; *Ri-1*), has a greater plant height, a larger seed size, and enhanced resistance to *Xoo* infection.

## Results

### Expression of *OsPR10a* is affected by *OsCDPK1*

**Figure 1:**
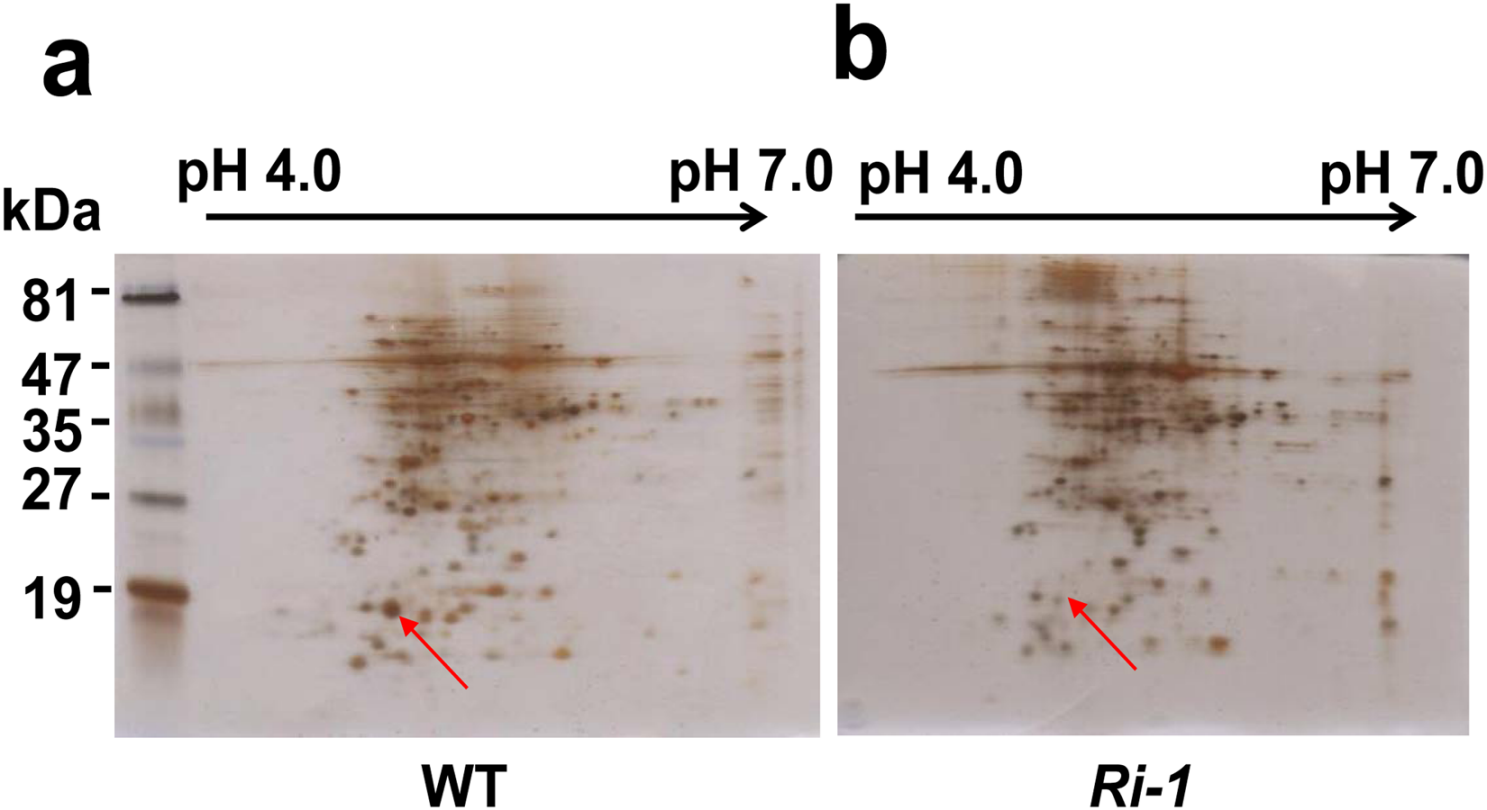
Two-dimensional gel electrophoresis (2-DE) analysis of changes in the protein levels in the WT and *Ri-1* seedlings. Total proteins were purified from 14-day-old seedlings, separated by 2-DE, and visualized by silver staining. The arrow indicates the OsPR10a protein spot that shows differences in the protein abundance between the WT (**a**) and *Ri-1* (**b**) plants.

The constitutively active mutants of CDPKs can bypass Ca^2+^ and stress signals and activate the expression of downstream responsive genes^1,7^. Therefore, we generated transgenic rice plants carrying a constitutively active truncated form of *OsCDPK1* (*OEtr-1*) under the control of the maize ubiquitin promoter and performed functional studies^27^. The *OEtr-1* transgenic rice seedlings showed a semi-dwarf phenotype, whereas the transformants subjected to the RNA interference gene knockdown (*Ri-1*) exhibited a slender-growth phenotype^27^. Two-dimensional gel electrophoresis was performed to isolate the proteins (or genes) regulated by *OsCDPK1*. Twenty up- or down-regulated candidate proteins were identified. Unexpectedly, among the differently regulated genes, *OsPR10a* (D38170), which encodes the pathogen resistance protein PBZ1, was highly expressed in WT seedlings, but only small amounts of the protein were detected in *Ri-1* seedlings (Fig. 1). Therefore, we postulated that *OsPR10a* might be positively regulated by *OsCDPK1*. To test this hypothesis, the third leaf of three-week-old WT, *OEtr-1* and *Ri-1* seedlings were inoculated with a scissor-contaminated *Xoo* using the leaf tip-clipped method^29^ and then grown for 1 d. Total RNA extracted from the treated leaves was purified and subjected to northern blot analysis. In the controls, a small amount of *OsPR10a* mRNA could still be observed in *OEtr-1*, while it was almost undetectable in WT and *Ri-1* (Supplementary Fig. S1a). After quantification of the hybridization signal by densitometer, the relative *OsPR10a* mRNA expression levels in the *OEtr-1* and *Ri-1* were 2.8- and 0.2-fold, respectively, when compared to the uninfected WT control (1.0-fold) (Supplementary Fig. S1b). In response to *Xoo* inoculation, the *OsPR10a* expression was strongly induced in *OEtr-1* (30.8-fold), moderately induced in the WT (20.4-fold), and fewer induction in *Ri-1* (11.2-fold), than that of the uninfected WT control (Supplementary Fig. S1). These results indicate that *OsCDPK1* may mediate the defense signal to activate *OsPR10a* expression under *Xoo* attack.

**Figure 2:**
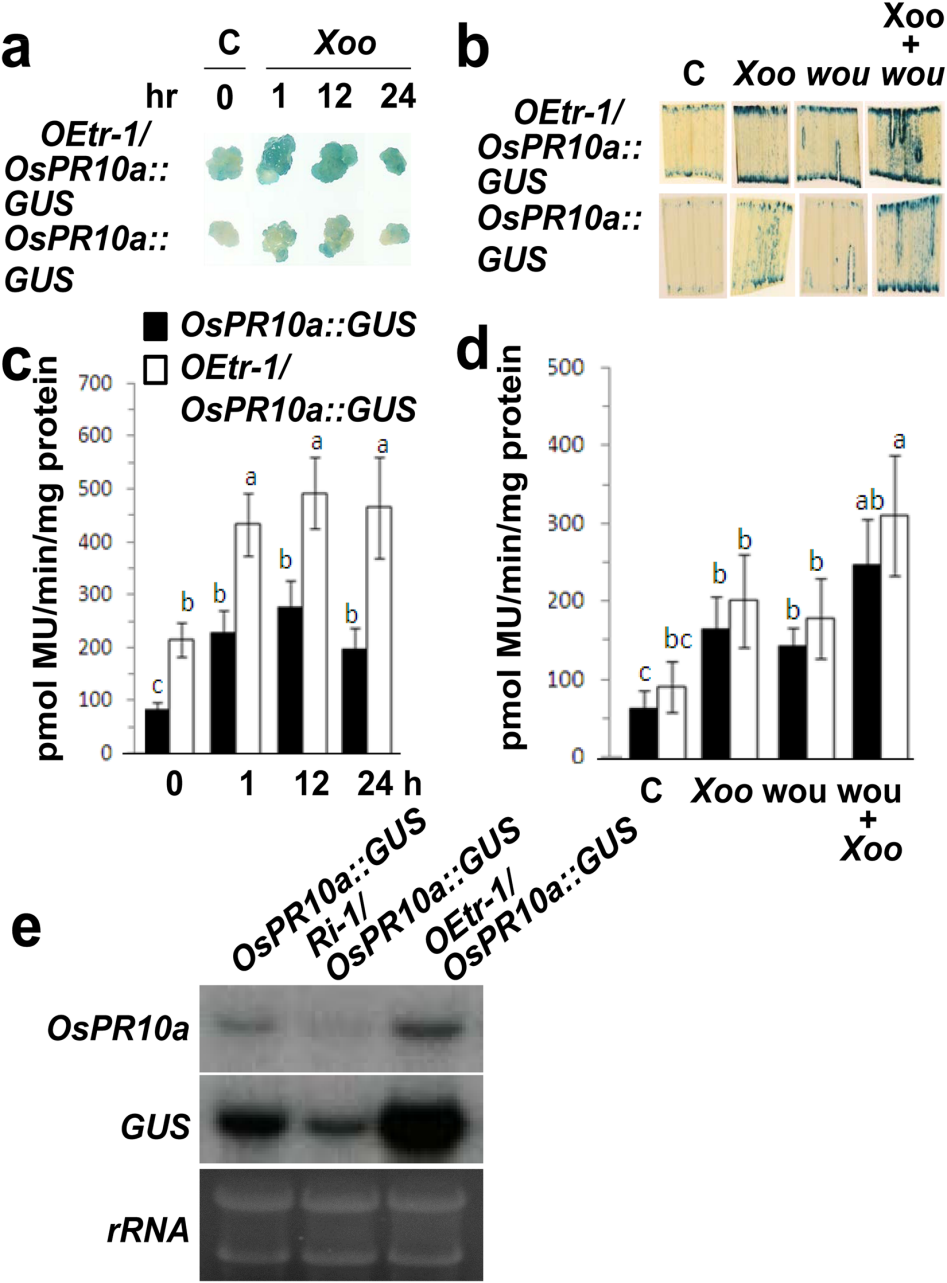
Histochemical staining of β-glucuronidase (GUS) activity in suspension-cultured cells and leaves from transgenic plants. (**a)** Suspension-cultured cells were inoculated with *Xanthomonas oryzae* pv. *oryzae* (*Xoo*) for 1, 12, and 24 h. Cells were harvested at the indicated time and stained with X-Gluc (5-bromo-4-chloro-3-indolyl-beta-D-glucuronic acid) for 2 h. (**b)** The third leaves of three-week-old seedlings were subjected to no treatment (control; C), spray-inoculation with *Xoo* (1.0 × 10^10^ CFU/mL) (*Xoo*), wounding with a sterilized razor blade (wou), or a concurrent treatment of wounding and spray-inoculation with *Xoo* (Wou + *Xoo*). One day post-inoculation, the treated leaves were cut, stained with X-Gluc for 12 h, and photographed. Fluorometric quantification of GUS activity in (**c)** suspension-cultured cells and (**d)** treated leaves using 4-MUG as the substrate. Different letters above the bars indicate significant differences as indicated by ANOVA (*P* < 0.05). The data are presented as the means ± SD (*n* = 12). (**e**) Northern blot analysis of *OsPR10a* and *GUS* gene expression in dihybrid plants following *Xoo* infection. Fourteen-day-old seedlings were wounded, spray-inoculated with *Xoo* (leaves were punctured with a needle before spraying) (1.0 × 10^10^ CFU/mL), and grown for 1 day. Total RNA from infected leaves was purified and subjected to northern blot hybridization using a probe prepared from an *OsPR10a-*specific region or GUS coding sequence. rRNAs served as the quantity control.

To verify the hierarchical relationship between *OsCDPK1* and *OsPR10a*, we integrated the transgenes *OEtr-1* and *OsPR10a::GUS* into a single genotype by crossing the *OEtr-1* and *OsPR10a::GUS* lines to generate a dihybrid plant designated *OEtr-1*/*OsPR10a::GUS*. A PCR analysis of DNA isolated from calli induced from F_2_ seeds confirmed that the progeny harbored both transgenes. Positive calli (i.e., calli that harbored both transgenes) and calli derived from the *OsPR10a::GUS* line were established as suspension cell cultures. The cells were then transferred to fresh medium or 50 mL culture medium containing 10 μL *Xoo* (1.0 × 10^10^/mL) and cultured for an additional 1, 12, and 24 h. Before and after *Xoo* inoculation, the GUS staining and the quantitative GUS activity assay both detected in the *OEtr-1*/*OsPR10a::GUS* line was higher than that in the *OsPR10a::GUS* line (Fig. 2a and c). Similarly, when the leaves in three-week-old plants were either treated with *Xoo* (spray-inoculation) or wounding, or concurrent treatment by wounding and *Xoo*, all the treated leaves in *OEtr-1*/*OsPR10a::GUS* have showed stronger GUS activity than those leaves from the *OsPR10a::GUS* plants (Fig. 2b,d). Moreover, many GUS staining foci were distributed throughout the *Xoo* spray-inoculated leaves with or without wounding in both examined lines (Fig. 2b), demonstrating that the spray inoculation successfully achieved *Xoo* infection. Furthermore, an additional dihybrid plant, *Ri-1/OsPR10a::GUS*, was further analyzed using northern blot hybridization. Total RNA was extracted from the *Xoo*-infected leaves in 14-d-old *OsPR10a::GUS*, *Ri-1/OsPR10a::GUS* and *OEtr-1/OsPR10a::GUS* seedlings and subjected to northern blot analysis. The mRNA level of both *OsPR10a* and *GUS* was lower in *Ri-1/OsPR10a::GUS* but higher in *OEtr-1/OsPR10a::GUS* than it was in *OsPR10a::GUS* (Fig. 2e). These results suggest that *OsCDPK1* is an upstream regulator of *OsPR10a* that positively affects its gene expression.

### Expression of *OsCDPK1* and *OsPR10a* is up-regulated in response to jasmonic acid, salicylic acid and *Xoo* treatments

**Figure 3:**
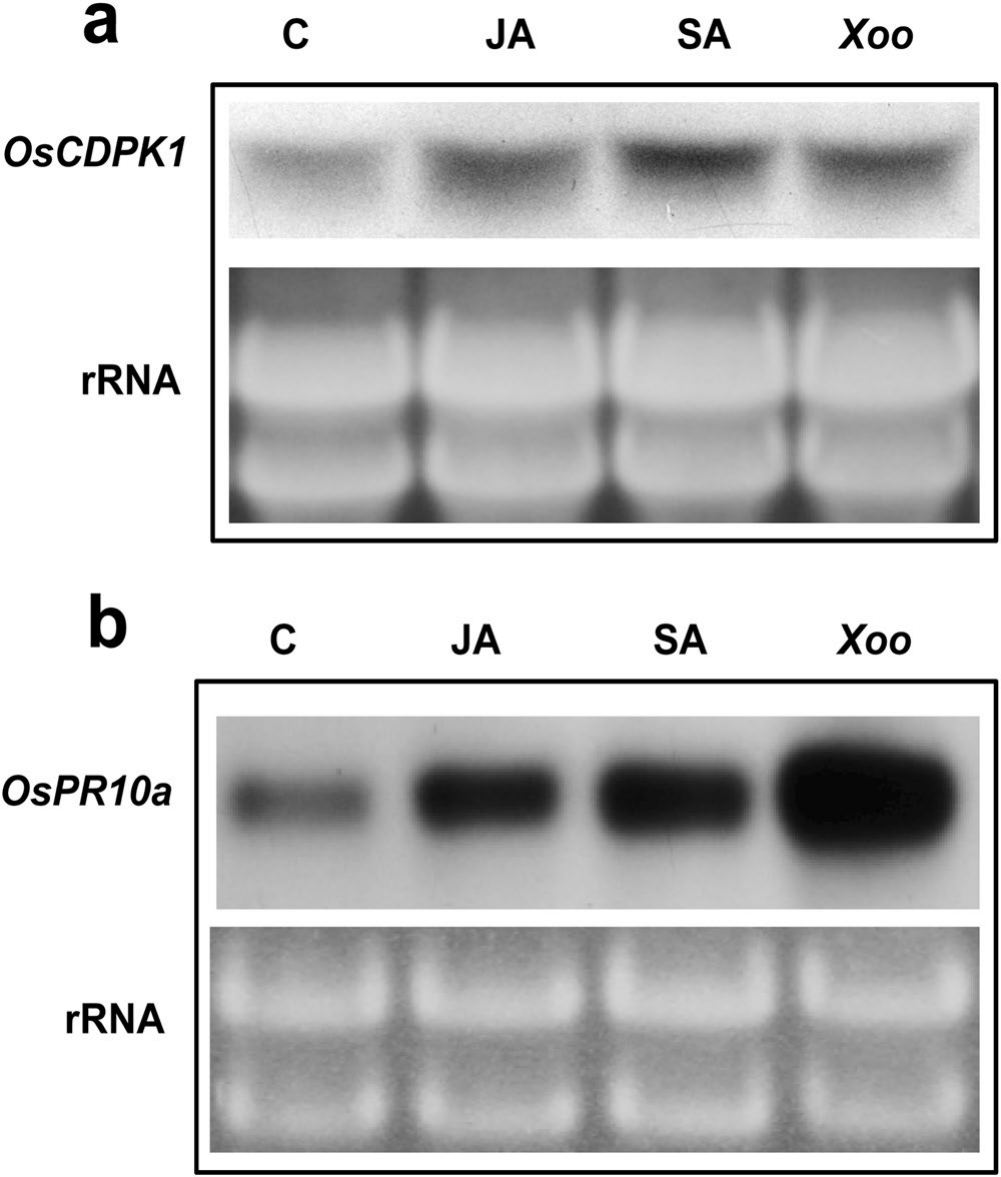
Expression of *OsCDPK1* and *OsPR10a* in response to salicylic acid (SA), jasmonic acid (JA) and *Xanthomonas oryzae* pv. *oryzae* (*Xoo*) treatments. The third leaves of three-week-old seedlings were wounded; spray-inoculated with *Xoo* (leaves were punctured with a needle before spraying) (1.0 × 10^10^ CFU/mL), JA (100 μM), or SA (100 μM); and grown for 1 d. Total RNA from the treated leaves was purified and subjected to a northern blot analysis using (**a)** *OsCDPK1*-specific or (**b)** *OsPR10a*-specific regions as probes.

The plant hormones jasmonic acid (JA) and salicylic acid (SA) play crucial roles in the defense signaling pathways. Therefore, we examined whether *OsCDPK1* expression was induced by pathogen infection alone or also by JA or SA. The third leaf of three-week-old WT, *OEtr-1* and *Ri-1* seedlings was sprayed with JA (100 μM) or SA (100 μM) or the leaf tips were wounded and then spray-inoculated with *Xoo* (1.0 × 10^10^ CFU/mL), followed by growth for 1 d. Total RNA was obtained from the treated leaves and subjected to northern blot analysis. We observed that the mRNA level of OsCDPK1 was higher following the JA, SA, and *Xoo* treatments than it was in the control (Fig. 3a), indicating that the expression of *OsCDPK1* was induced not only by *Xoo* but also by JA and SA. If *OsCDPK1* acts as an upstream component of pathogen signaling and activates *OsPR10a* expression, the expression of *OsPR10a* should correspond to that of *OsCDPK1* following the JA, SA and *Xoo* treatments. As shown in Fig. 3b, we also observed abundant expression of *OsPR10a* under JA, SA, and *Xoo* treatments. Similarly, slot-blot analysis reconfirmed the expression of both genes was induced by the JA and SA treatments and further increased following the combined exogenous application of JA (100 μM) and SA (100 μM) to the plants (Supplementary Fig. S4). These results suggested that *OsCDPK1* may act as a mediator in the JA and SA signaling pathways, thereby enhancing *OsPR10a* gene expression.

**Figure 4:**
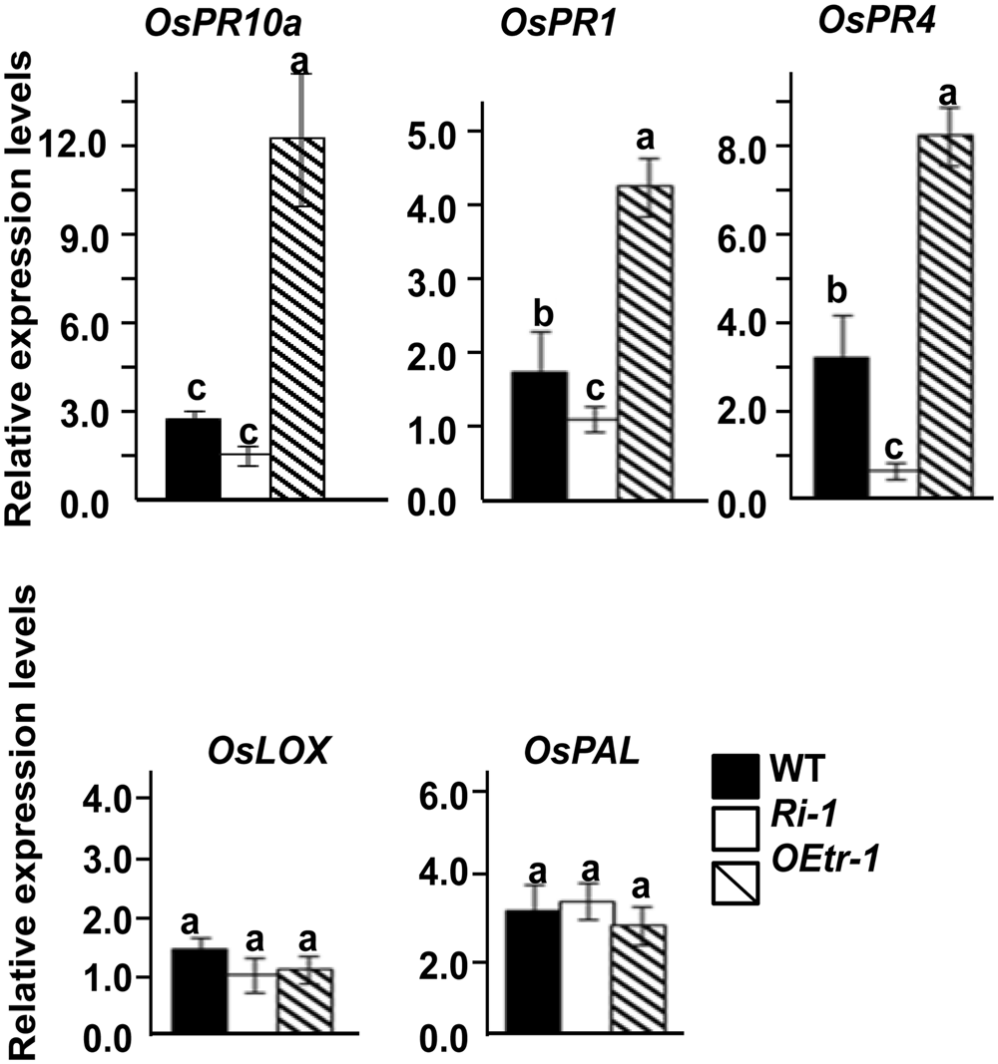
Expression of pathogenesis-related genes in WT, *Ri-1* and *OEtr-1* seedlings inoculated with *Xanthomonas oryzae* pv. *oryzae*. The tip of the third leaf of the three-week-old seedlings was wounded with a needle and then spray-inoculated with *Xoo* (1.0 × 10^10^ CFU/mL). One day after the inoculation, total RNA was isolated from the treated leaves and subjected to a real-time RT-PCR analysis. Quantification of the relative expression levels of the selected genes normalized to the expression level of the internal control, i.e., *OsActin*. All reactions were analyzed in three replicates. Different letters above the bars indicate significant differences as indicated by ANOVA (*P* < 0.01). The data are presented as the mean ± SD of three independent repeats. *OsPR10a*: a ribonuclease-like pathogenesis-related gene; *OsPR1*: an antifungal protein family member; *OsPR4*: a chitinase gene; *OsLOX*: lipoxygenase gene; *OsPAL*: a phenylalanine ammonia lyase gene. Primer sets and gene accession numbers are listed in Supplementary Table S1.

To further examine the possible roles of *OsCDPK1* in the response to JA and SA signaling, the PR-related genes *OsPR1* (encoding β-1,3-glucanase) and *OsPR4* (encoding a member of the chitinase family), which are known as SA- and JA-signaling responsive marker genes^29^, respectively, and the genes *OsLOX* (encoding lipoxygenase) and *OsPAL* (encoding phenylalanine ammonia lyase), which are key enzymes in the JA and SA biosynthesis pathways^30,31^, were subjected to a real-time RT-PCR analysis. As shown in Fig. 4, compared with the levels observed in the WT plants, the relative expression levels of *OsPR1*, *OsPR4*, and *OsPR10a* were significantly up-regulated (P < 0.01) in the *OEtr-1* lines but down-regulated in the *Ri-1* plants, whereas the expression of *OsLOX* and *OsPAL* was unaffected. These results demonstrate that *OsCDPK1* is involved in the innate immunity in rice and regulates a broad spectrum of PR-related genes involved in disease resistance.

In addition, we examined the expression of *OsCDPK1* and *OsPR10a* in response to pathogen infection in germinating rice seeds. Transgenic seeds derived from the reporter lines *OsCDPK1::GUS* and *OsPR10a::GUS* ^28^ (Supplementary Fig. S5a) were germinated on ½ MS medium for 1–5 days and then stained to assess GUS activity. Compared with the germination in the healthy seeds, *GUS* was specifically expressed in the embryo and shoot, but not in the endosperm, in both transgenic lines (Supplementary Fig. S5b). Furthermore, we found that GUS staining was induced in the zone surrounding the brown spot lesions in the germinating seeds infected with an unidentified pathogen, but the staining was not visible around the spot lesions on the endosperm of the *OsActin::GUS* line (Supplementary Fig. S5c). These results demonstrate that *OsCDPK1* and *OsPR10a* displayed similar gene expression patterns in the germinating rice seeds and were specifically induced by the pathogen infection.

### Overexpression and RNA interference of *OsCDPK1* enhanced and reduced pathogen resistance, respectively, in transgenic rice

**Figure 5:**
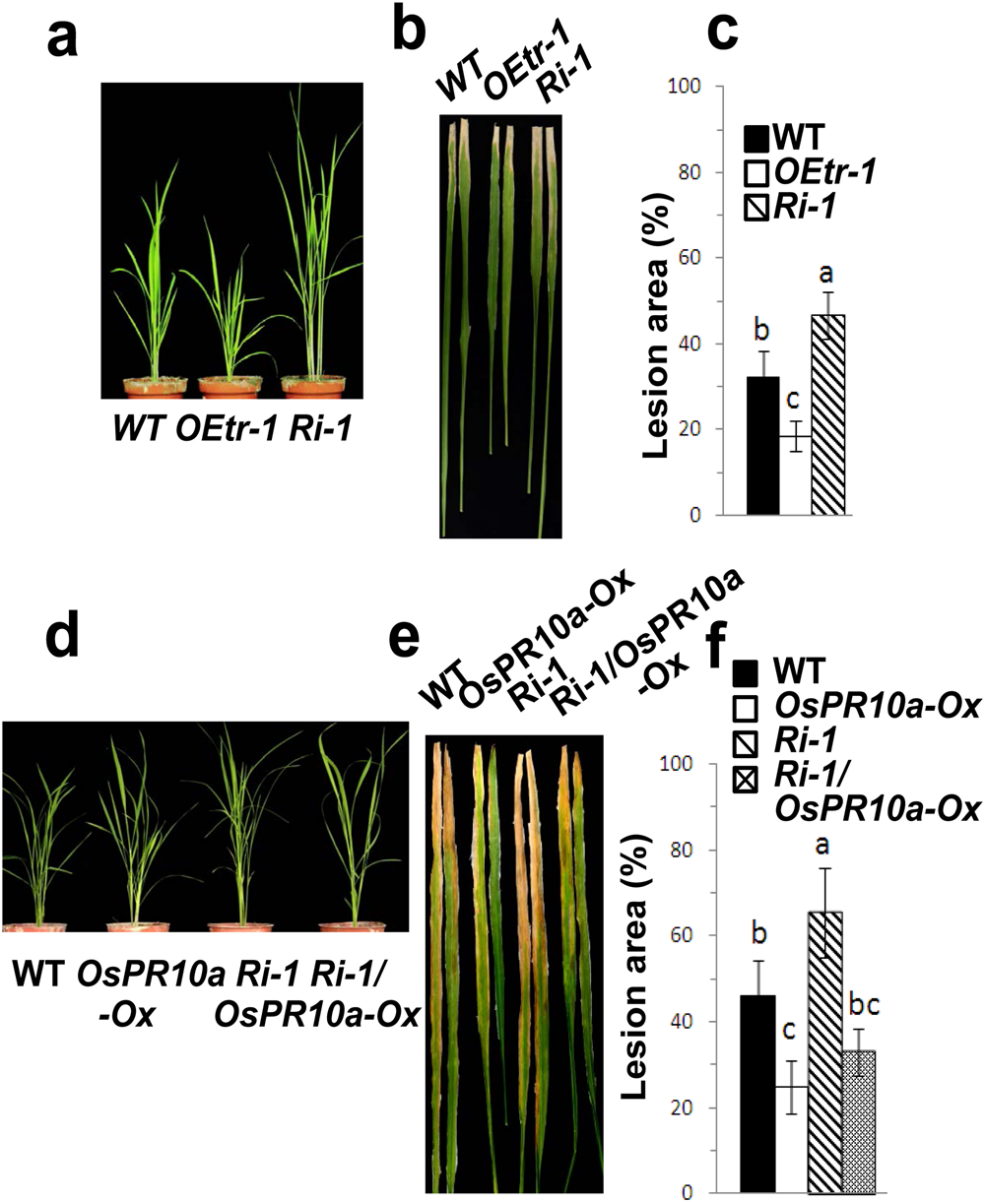
Ectopic expression of *OsCDPK1* in rice and the dihybrid transgenic plant *Ri-1*/*OsPR10a-Ox* in response to *Xanthomonas oryzae* pv. *oryzae* infection. (**a)** and (**d)** Phenotypical comparison of three-week-old wild-type (WT) and transgenic seedlings. (**b)** and (**e)** The tip of the third leaf of three-week-old plants was excised with a razor blade contaminated with *Xoo* (1.0 × 10^10^ CFU/mL). Infected plants were incubated in a growth chamber for disease development. Photographs were obtained (**b)** 10 days or (**e)** 20 days after the inoculation. (**c)** and (**f)** Quantification of the lesion area. The experiments were repeated three times. Different letters above the bars indicate significant differences as indicated by ANOVA (*P* < 0.05). The data are presented as the means ± SD (*n* = 12).

The present data reveal that *OsCDPK1* positive affects *OsPR10a* and that the expression of *OsCDPK1* is induced by SA, JA, and *Xoo* treatments. Moreover, *OsPR10a*-overexpressing rice plants exhibit increased resistance to *Xoo* infection^28^. To determine whether *OEtr-1* plants show increased resistance to pathogen attack, *OEtr-1* and *Ri-1* plants were inoculated with *Xoo*. A scissor contaminated with *Xoo* (1.0 × 10^10^ CFU/mL) was used to excise the apical 1-cm portion of the third leaf of three-week-old seedlings (Fig. 5a). At 10 DPI (days post-inoculation), the lesion area in the WT, *OEtr-1* and *Ri-1* leaves was 32 ± 6.1%, 18 ± 3.3%, and 46 ± 7.2%, respectively (Fig. 5b and c), revealing that the *OEtr-1* plants had improved resistance to *Xoo* infection. To examine disease response in the whole plants, *Xoo* inoculation was performed as described in the Methods. Before *Xoo* inoculation, plants from the three tested lines were healthy, and the *Ri-1* plants were taller while the *OEtr-1* plants were shorter than WT plants (Supplementary Fig. S6a), this result is consistent with our previous finding in which OsCDPK1 was shown to act as a negative regulator of gibberellin (GA) biosynthesis^27^. At 10 DPI, the leaves of the *Ri-1* and WT seedlings developed chlorotic or necrotic lesion symptoms, which developed earlier in the *Ri-1* seedlings, but these lesions were barely detectable in the *OEtr-1* seedlings (Supplementary Fig. S6a). At 15-20 DPI, an increase in the severity of the symptoms was observed in the *Ri-1* seedlings, and up to 78–88% of all leaves became chlorotic or showed necrotic lesions with subsequently wilting and dying leaves, whereas only approximately 20–26% of the *OEtr-1* seedlings displayed chlorotic symptoms (Supplementary Fig. S6a). At 20 DPI, the survival rate of the WT, *OEtr-1*, and *Ri-1* seedlings was 21 ± 3.1%, 74 ± 10.3%, and 12 ± 3.2%, respectively (Supplementary Fig. S6b). These results indicated that OsCDPK1 enhances pathogen resistance in rice.

### Enhanced resistance to bacterial blight disease and seed size in the dihybrid transgenic rice *Ri-1*/*OsPR10a-Ox*

The *OsCDPK1*-silenced rice plants produce larger seeds and an increased crop yield^27^ but are more susceptible to pathogen infection (Fig. 5b and c; Supplementary Fig. S6). The present results demonstrate that *OsCDPK1* acts as a positive regulator of *OsPR10a*. Therefore, we hypothesize that the overexpression of *OsPR10a* in the *Ri-1* plants might lead to the development of a novel rice genotype with an improved crop yield and disease resistance. The *Ri-1* line was crossed with *OsPR10a-Ox7* ^28^ (named *OsPR10a-Ox* in the present study) to generate the *Ri-1/OsPR10a-Ox* line. The genotypes of the F_2_ plants were examined by Southern blot hybridization using *Hpt* as a probe (Supplementary Fig. S7), which confirmed that the dihybrid plants harbored both transgenes. To evaluate the difference in disease resistance among the WT, *OsPR10a-Ox*, *Ri-1*, and dihybrid line *Ri-1/OsPR10a-Ox*, the plants were inoculated with *Xoo* by clipping the leaf tip as previously described. At 20 DPI, the leaves of the WT and *Ri-1* plants had developed large lesions, but the leaves of the *OsPR10a-Ox* and *Ri-1/OsPR10a-Ox* lines had developed smaller lesions (Fig. 5e). The average lesion area in the WT, *OsPR10a-Ox*, *Ri-1*, and *Ri-1/OsPR10a-Ox* plants was 46 ± 10.3%, 25 ± 6.7%, 65 ± 11.4%, and 33 ± 5.2%, respectively (Fig. 5f). These results indicate that the dihybrid rice *Ri-1/OsPR10a-Ox* might also have improved disease resistance.

**Figure 6:**
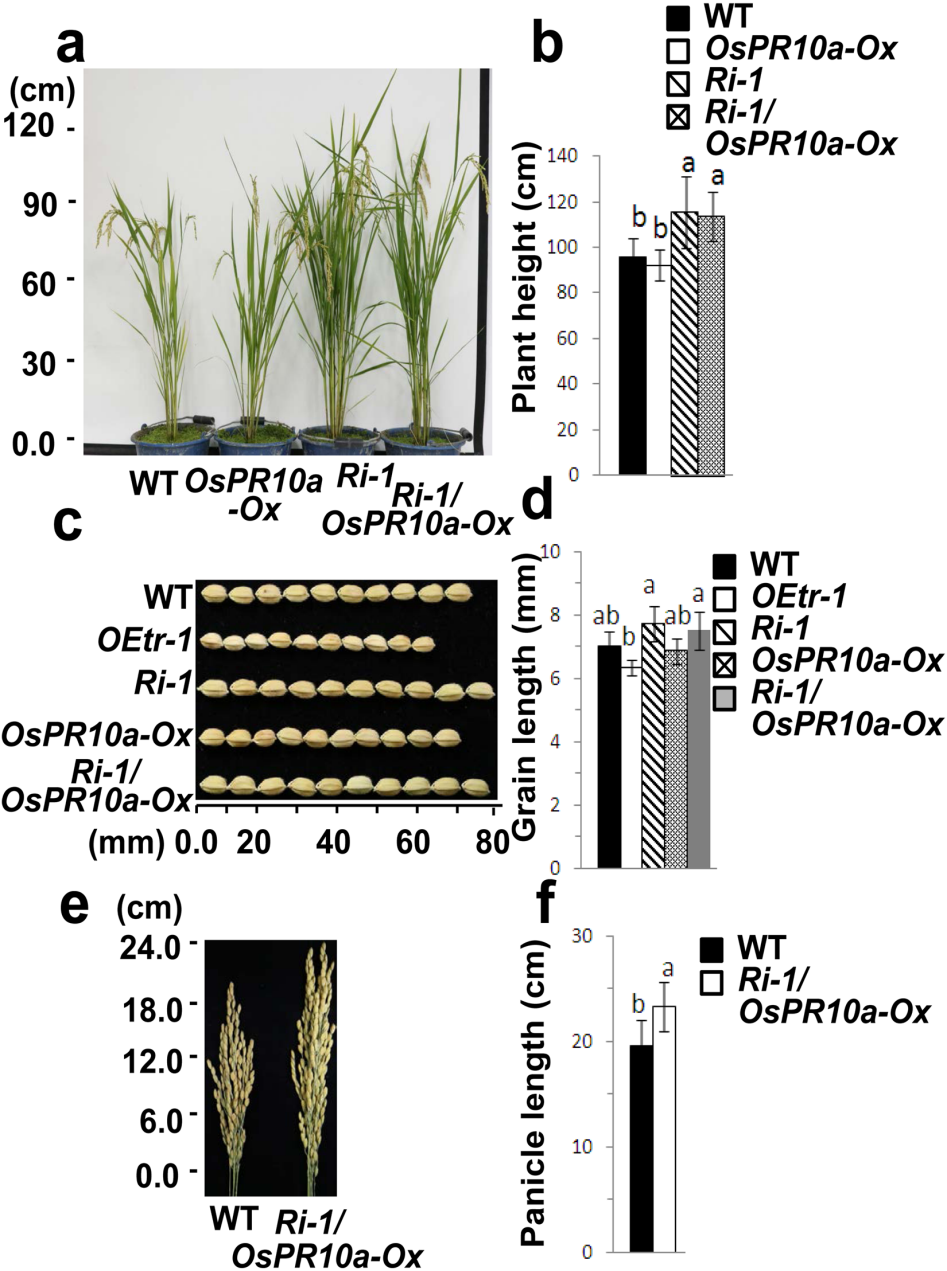
Phenotypes of *OsPR10a-Ox*, *Ri-1*, the dihybrid *Ri-1/OsPR10a-Ox*, and wild-type (WT) rice. (**a)** Fourteen-day-old seedlings were transplanted individually into buckets. Mature plants (WT, *OsPR10a-Ox*, *Ri-1*, and *Ri-1/OsPR10a-Ox*) at the middle heading stage (98 days) were photographed. (**b)** Quantification of plant height. (**c)** Comparison of seed size between the WT and transgenic lines. (**d)** Quantification of grain length. (**e**) Panicle phenotypes of the WT and *Ri-1/OsPR10a-Ox*. Three panicles were collected from the WT and dihybrid plants and photographed. (**f)** Quantification of panicle length. (**b**, **d** and **f**) Different letters above the bars indicate significant differences as indicated by ANOVA (*P* < 0.05). The data are presented as the means ± SD (*n* = 20).

We also examined resistance to *Xoo* infection by performing a whole-plant experiment. Three-week-old seedlings were inoculated with *Xoo* as described in Supplementary Figure S8. Before *Xoo* infection, all plants showed healthy phenotypes, and the *Ri-1* and *Ri-1/OsPR10a-Ox* plants were taller than the WT and *OsPR10a-Ox* plants (Supplementary Fig. S8a, upper panel). The *Ri-1/OsPR10a-Ox* plants inherited the slender-growth phenotype from the *Ri-1* line^27^. At 20 DPI, severe disease symptoms were observed in the WT and *Ri-1* plants, which was consistent with the results shown in Supplementary Figure S6, whereas only weak chlorosis was detected in the *OsPR10a-Ox* and *Ri-1/OsPR10a-Ox* seedlings (Supplementary Fig. S8a, middle panel). At 25 DPI, most WT (78%) and *Ri-1* (86%) seedlings were necrotic and subsequently died, whereas only a few leaves were chlorotic in the *OsPR10a-Ox* and *Ri-1/OsPR10a-Ox* lines (Supplementary Fig. S8a, lower panel). Thus, the latter two lines exhibited strongly enhanced resistance to *Xoo* infection. The survival of the WT, *OsPR10a-Ox*, *Ri-1*, and *Ri-1/OsPR10a-Ox* seedlings at 25 DPI was 22 ± 6.4%, 68 ± 12.2%, 14 ± 5.5%, and 66 ± 16.4, respectively (Supplementary Fig. S8b). These results provide direct evidence indicating that the dihybrid rice *Ri-1/OsPR10a-Ox* can overcome the effect of *OsCDPK1* silencing on the susceptibility to *Xoo* infection and achieve improved disease resistance. Furthermore, our previous studies demonstrated that the transgenic rice *Ri-1* has enhanced seedling growth and produces larger seeds^27^, and no significant differences in seed size, seed set and seedling development were observed between the WT and *OsPR10a*-overexpressing rice plants^28^. The present results show that the dihybrid rice *Ri-1/OsPR10a-Ox*, which exhibits a phenotype similar to that of *Ri-1*, also has an increased plant height (Fig. 6a and b), grain size (Fig. 6c and d), and panicle length (Fig. 6e and f). The mature plant height of *Ri-1/OsPR10a-Ox* was 17.3% higher than that of the WT plants (Fig. 6b). Compared to the WT, the *Ri-1/OsPR10a-Ox* line showed an 8.3% increase in grain length (Fig. 6d) and a 15.8% increase in panicle length (Fig. 6f). These results demonstrate that the transgenic dihybrid rice *Ri-1/OsPR10a-Ox* has a potential to increase disease resistance and improve grain yield in rice.

## Discussion

The overexpression of *OsPR10a* in rice confers increased resistance to *Xoo* bacterial blight^28^. The *OsCDPK1*-silenced plants (*Ri-1*) produced larger seeds (Fig. 6), but the grain number was unaffected; therefore, these plants provided an improved crop yield^27^ but were more susceptible to the pathogen challenge (Fig. 5). In the current study, using a gene pyramiding approach to combine *OsCDPK1* silencing and *OsPR10a* overexpression, we generated dihybrid plants that retained the higher crop yield trait of *Ri-1* and not only overcame the sensitivity to pathogen infection but also showed enhanced disease resistance with a phenotype similar to that of *OsPR10a-Ox*.

Despite the importance of *OsPR10a* in the resistance to pathogen infection in rice, its regulatory pathway remains poorly understood. In the present study, we show that the protein kinase *OsCDPK1* functions as a positive effector of *OsPR10a* in rice. Both loss-of-function and gain-of-function experiments were performed to support the role of *OsCDPK1* in defense responses and the regulation of *OsPR10a* expression. These results support the hypothesis that OsCDPK1 acts as an upstream signal transducer in the rice defense response to a pathogen challenge. The plant hormone SA is known to play key roles in plant defense against biotrophic and hemi-biotrophic pathogens^31^, and JA is involved in resistance to necrotrophic pathogens and herbivorous insects^31^. The SA and JA signaling pathways interact antagonistically to regulate responses to different biotic stresses^30,32–35^. However, a few studies have reported that SA and JA may have a synergistic effect in defense signaling at low concentrations despite their seemingly antagonistic effects at high concentrations (i.e., SA ≥ 350 μM and JA ≥ 125 μM)^36,37^. *OsPR10a* (*PBZ1*) is induced by exogenous SA, JA, *Xoo*, and the blast fungus *Magnaporthe grisea* ^28,38–40^, suggesting that *OsPR10a* might confer broad-spectrum resistance to pathogens. These findings are consistent with the present results, and we showed that both *OsCDPK1* and *OsPR10a* were synergistically up-regulated by the treatment with SA (100 μM) and/or JA (100 μM) or infection by the biotrophic pathogen *Xoo* (Fig. 3; Supplementary Fig. S4). In addition, we observed that the SA and JA signaling responsive genes *OsPR1* and *OsPR4* ^30^, respectively, were both induced in *OEtr-1* but repressed in *Ri-1*, and the expression of *OsPAL* and *OsLOX*, which are key enzymes in the SA and JA biosynthesis pathways, respectively^31,32^, did not significantly differ (P < 0.01) among the WT, *OEtr-1*, and *Ri-1* (Fig. 4). These results suggest that *OsCDPK1* might mediate the SA and JA signaling pathways, thereby inducing *OsPR10a* expression and resulting in broad-spectrum disease resistance in rice. Moreover, the GUS activity staining and its gene expression were considerable enhanced and reduced in the *OEtr-1/OsPR10a::GUS* and *Ri-1/OsPR10a::GUS*, respectively, in response to wounding and *Xoo* infection (Fig. 2), indicating that *OsCDPK1* acts upstream of *OsPR10a* in rice defense signaling pathways upon pathogen attack and wounding. These results suggest that *OsCDPK1* might phosphorylate its novel substrate thereby directly or indirectly regulates *OsPR10a* expression via an unknown transcription factor binding to the *OsPR10a* promoter. The ChIP (chromatin immunoprecipitation) cloning strategy therefore can be used to isolate this specific transcription factor to unravel the mechanism of *OsCDPK1*-mediated *OsPR10a* expression, to clarify the effect of *OsCDPK1* on regulation of *OsPR10a* is through the direct or by indirect pathways. It is worth noting that *OsPR10a* was also expressed in response to *Xoo* infection in the *Ri-1* plants (Supplementary Fig. S1), although at a lower level than that in the WT, suggesting that other signaling pathways may contribute to the regulation of *OsPR10a* expression following *Xoo* infection. Two rice transcription factors, i.e., OsWRKY6 and OsWRKY51, can bind to the *OsPR10a* promoter and activate its transcription in response to *Xoo* inoculation^41,42^. Moreover, overexpression of OsWRKY45–2 also induces *OsPR10a* expression in rice^22^. Collectively, these findings suggest that certain *OsCDPK1*-dependent and *OsCDPK1*-independent signaling pathways may coordinate the regulation of *OsPR10a* expression in response to *Xoo* attack.

**Figure 7:**
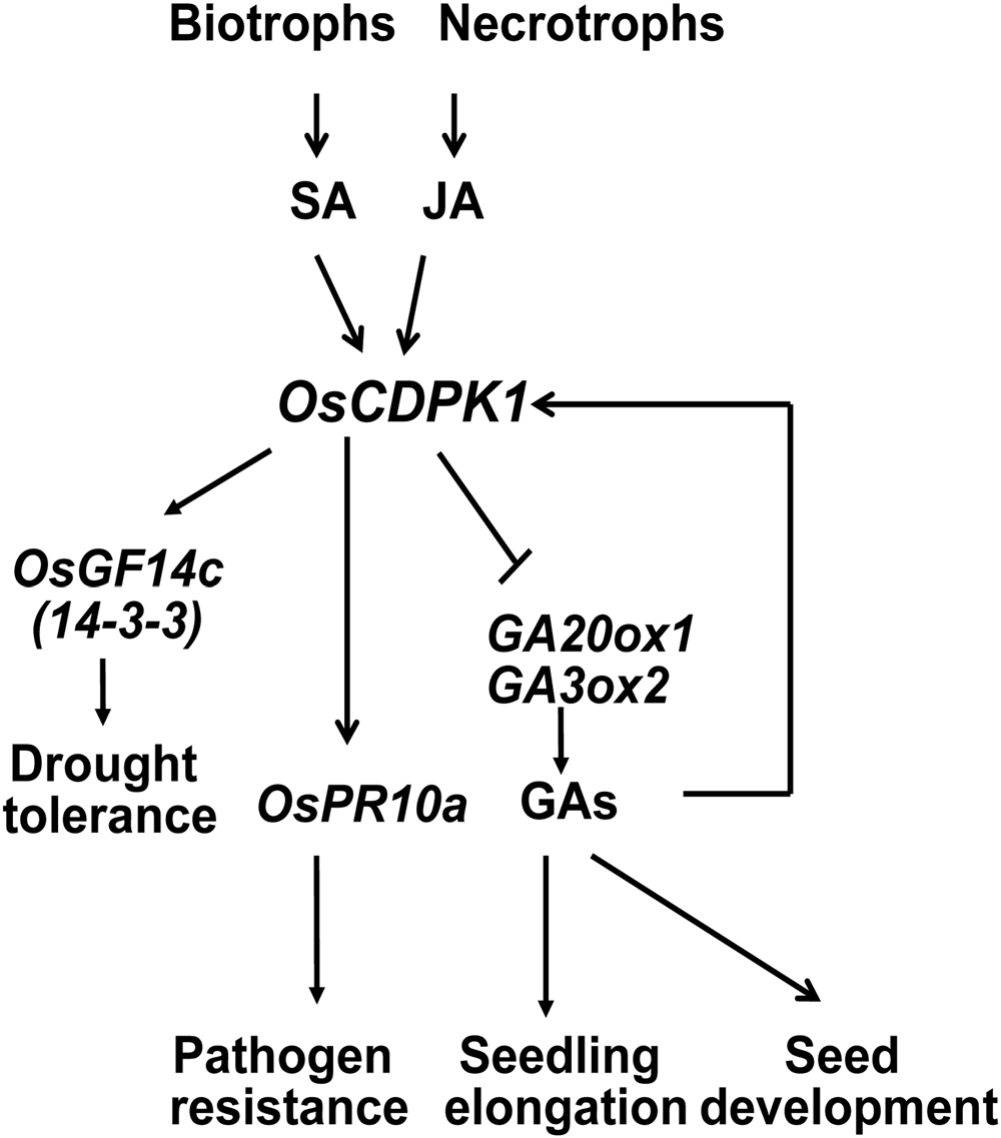
Proposed roles of *OsCDPK1* in the interconnection of signaling pathways to coordinate responses to biotic and abiotic stresses and plant developmental processes. Details of the model are described in the text.

In plants, the CDPKs play diverse roles in response to biotic and abiotic stresses and modulate various aspects of plant growth and development. Our results reveal that OsCDPK1 performs multiple functions in response to biotic and abiotic stresses and developmental regulation. The *OsCDPK1*-overexpressing rice (*OEtr-1*) shows enhanced tolerance to drought stress and negatively regulated GA biosynthesis, resulting in a semi-dwarf seedling phenotype^27^. Based on our current and previous studies, we propose a model in which *OsCDPK1* plays vital roles in the interconnection of various signaling pathways to coordinate the physiological adaptive responses to biotic and abiotic stresses and adverse growing conditions (Fig. 7). The plant signaling pathways involved in the stress response and growth are generally antagonistic to each other, in which a few regulators play key roles in the cross-talk and mediation of the balance between stress response and development^35^. When plants are subjected to biotic and abiotic stresses, the stress-signaling networks induce an increase in the levels of certain phytohormones, such as SA, JA, abscisic acid (ABA), and ethylene, and subsequently induce the expression of stress-related genes. In contrast, these networks have a negative effect on plant growth-promoting hormones, such as GAs, auxin, and cytokinin, thus resulting in the attenuated expression of development-related genes^43–50^. For example, in *Arabidopsis*, the GA signaling repressor DELLA increases the sensitivity to biotrophic pathogens and resistance to necrotrophic pathogens by orchestrating the relative signaling strength of SA and JA^51^. DELLA competes with MYC2 (a transcriptional activator of JA signaling) for binding to JASMONATE-ZIM DOMAIN (JAZ; a key repressor of JA signaling), which results in the release of MYC2 and activates the JA signaling response^52–54^. These findings demonstrate that the JA signaling pathways may be compromised by GA through the degradation of DELLA. Therefore, DELLA might act as a key regulator of the crosstalk among the GA, SA, and JA signaling pathways. Moreover, SA signaling is induced by infection with virulent biotrophs, which simultaneously weakens ABA signaling, indicating that antagonistic interactions likely occur between the biotic and abiotic signaling pathways^45,55^. These results reveal that plants have developed multiple mechanisms to coordinate a variety of hormone signals to modulate the balance between biotic and abiotic stresses and growth responses.

Generate crops with stress tolerance and (or) resistance and a high yield using traditional breeding methods is challenging due to the antagonistic regulatory pathways involved in stress responses and developmental processes^48^. Thus, genetic modification strategies might be useful for achieving this aim, but an understanding of the essential genes involved in the antagonistic pathways in individual plants is essential. We have shown that OsCDPK1 enhances drought tolerance mediated by GF14c^27^ and increased resistance to *Xoo* infection by affecting *OsPR10a* expression (Figs 1, 2, 4, and 5). OsCDPK1 also confers a negative feedback loop to regulate GA biosynthesis by down-regulating *GA20ox1* and *GA3ox2* expression; therefore, *OEtr-1* plants have semi-dwarf seedlings and smaller grain phenotypes^27^ (Fig. 5). We also observed that *Ri-1* has an increased plant height and produces larger seeds but is more sensitive to *Xoo*. Using hybridization to combine the favorable traits of *Ri-1* and *OsPR10a-ox* in a single genotype, we demonstrated that the dihybrid transgenic rice *Ri-1/OsPR10a-ox*, which bypasses the sensitivity to *Xoo*, not only shows enhanced resistance to *Xoo* but also increased plant height and grain size. These results provide insight that improves our understanding of the molecular mechanisms underlying the balance between growth and defense responses in plants, which may be beneficial for traditional breeding and biotechnological approaches or the recently proposed ‘molecular strengthening’ (MOST) strategy^56^ to simultaneously improve stress tolerance and (or) resistance and increase crop yield in rice.

## Methods

### Plant material and preparation of cell suspension cultures

Immature seeds of the rice cultivar ‘Tainung 67’ were used for callus induction as described previously^27^. After incubation for about 30–40 days, the calli derived from the scutellum were transferred to liquid MS^57^ complete medium (MS salts containing 3% sucrose and supplemented with 10 µM 2,4-dichlorophenoxyacetic acid) to establish a suspension cell culture.

### Southern and northern and slot blot analyses

Genomic DNA or total RNA were isolated from three-week-old seedlings using urea extraction buffer or TRIzol reagent (Invitrogen, Carlsbad, CA, USA), respectively. DNA and RNA gel-blot analyses were conducted as described previously^27^. Ten micrograms of genomic DNA and total RNA were analyzed in 0.8% and 1.0% agarose gel, respectively, then transferred to a nylon membrane and hybridized with a digoxigenin*-*11*-*dUTP (DIG*-*11*-*dUTP) labelled probe. The blot was visualized using autoradiography with X-ray film.

### Protein extraction and two-dimensional gel electrophoresis analysis

Total proteins were extracted from 14-day-old seedlings (wild type [WT] and *Ri-1*) in an extraction buffer and mixed with an equal volume of phenol (pH 7.5). The aqueous supernatant was precipitated with acetone, the protein pellet was washed with acetone and air-dried, and stored at −80 °C. For first-dimension analysis, the isolated protein was rehydrated with rehydration buffer and analyzed using immobilized pH gradient (IPG) strips with pH 4–7 in accordance with the manufacturer’s instructions (Bio-Rad, Richmond, CA, USA). The second dimension was carried out using sodium dodecyl sulfate–polyacrylamide gel electrophoresis and the separated protein spots were visualized by silver staining. The candidate proteins were subjected to in-gel tryptic digestion and the samples were purified and subjected to liquid chromatography–tandem mass spectrometry as described previously^28,58^.

### Primers

The sequence of all primers used for PCR and real time RT-PCR amplification are listed in Supplementary Table S1.

### Construction of expression vectors

The plasmid constructs for generation of the transgenic plants *OEtr-1* (overexpression of the constitutively active truncated form of *OsCDPK1*), *Ri-1*, *OsPR10a-Ox* and *OsPR10a::GUS* were constructed as described previously (Supplementary Fig. S5a)^27,28^. To construct the *OsCDPK1::GUS* expression vector, a 2.0-kb DNA fragment containing the promoter and 5′-untranslated region of *OsCDPK1* (Supplementary Fig. S5a) was amplified by PCR using the primers *OsCDPK1P-FP* (5′-ATCCTGCAGTCTTATTAGGTAAGGCCTTG-3′) and *OCDPK1P-RB* (5′- ACTGGATCCAAGAACTCCTTATGCAAACC-3′). This DNA fragment was digested with *Pst*I and *Bam*HI, and cloned into the *GUS* expression vector pBX-2 as described previously^59^. The recombinant construct was then inserted into the *pSMY1H* binary vector^57^.

### Plant transformation

Rice calli were transformed using *Agrobacterium*-mediated gene transformation as previously described^57^.

### Real-time RT-PCR

The tips of the third leaf from three-week-old T_2_ seedlings were wounded with a needle and then spray-inoculated with *Xoo* (1.0 × 10^10^ CFU/mL). One day after the inoculation, the total RNA was isolated from the treated leaves using TRIzol reagent (Invitrogen), and the contaminated DNA was eliminated using a TURBO DNA-free kit (Ambion). Five micrograms of total RNA were used to synthesize first strand cDNA by M-MuLV reverse transcriptase (New England Biolabs) with oligo (dT) primer. Quantitative real time RT-PCR was performed with the Eco Real-Time PCR System (Illumina Inc., San Diego, CA) according to the manufacturer’s instructions. The gene-specific primer sets (Table S1) localized at the 3′-untranslated regions of each examined genes were used to evaluate the expression levels of *OsPR1*, *OsPR4*, *OsPR10a*, *OsLOX* and *OsPAL* in WT, *OEtr-1* and *Ri-1*. The relative expression levels were normalized to the expression of the internal control, i.e., *OsActin*. All reactions were analyzed in three replicates.

### Histochemical staining of GUS activity in rice cells and leaves

To stain the GUS activity in the cultured cells, the cell suspensions were cultured in liquid MS medium for 3 days, followed by inoculation with *Xoo* (1.0 × 10^8^ CFU/50 mL). The cells were collected at 0 (before inoculation; control), 1, 12, and 24 h after the inoculation for the GUS staining. To assay GUS activity in the leaves, the third leaf of three-week-old seedlings was untreated (control), treated with a spray-inoculation with *Xoo* (1.0 × 10^10^ CFU/mL), wounded with a razor blade contaminated with *Xoo* and then sprayed with *Xoo*, or wounded with a sterilized razor blade. The leaves were incubated in a growth chamber at 28 °C with > 80% relative humidity under a 16-h light/8-h dark photoperiod for 1 d. The treated leaves were cut, stained with X-Gluc (5-bromo-4-chloro-3-indolyl-beta-D-glucuronic acid; Sigma-Aldrich, St Louis, MO, USA) for 12 h, and then photographed. The fluorometric quantification of GUS activity was conducted according to the manufacturer’s instructions.

### Inoculation of rice seedlings with *Xoo*

To analyze the lesion area in the *Xoo*-infected leaves, the third leaf of three-week-old seedlings of the tested lines was inoculated with *Xoo* by excising the leaf tip with a scissor contaminated with *Xoo* (1.0 × 10^10^ CFU/mL)^29^. For the *Xoo* inoculation of whole plants, the tip of every leaf on three-week-old seedlings was penetrated at five different sites per leaf with a *Xoo*-contaminated needle and then spray-inoculated with *Xoo* (1.0 × 10^10^ CFU/mL) once daily for five days. The inoculated plants were incubated in a growth chamber at 28 °C, 90% relative humidity, under a 16 h light/8 h dark photoperiod for the development of the disease symptoms. The disease symptoms were evaluated by measuring the area of necrotic lesions on the leaves (expressed as a percentage of the total leaf area) or assessing the percentage survival of infected plants.

## Electronic supplementary material

Supplementary information
