# Supplementary material for: Overexpression of a constitutively active truncated form of OsCDPK1 confers disease resistance by affecting OsPR10a expression in rice
Source: Sci Rep. 2018 Jan 10;8:403. doi: 10.1038/s41598-017-18829-2 (PMC5762881; doi:10.1038/s41598-017-18829-2)
Supplement: Supplementary file 1 — Supplementary information [file 41598_2017_18829_MOESM1_ESM.pdf]

Supplementary information

Overexpression of a constitutively active truncated form of *OsCDPK1* confers disease resistance by affecting *OsPR10a* expression in rice

Siou-Luan He, Jian-Zhi Jiang, Bo-Hong Chen, Chun-Hsiang Kuo, Shin-Lon Ho*

Department of Agronomy, National Chiayi University, Chiayi 600, Taiwan

*Author for correspondence

Table S1 Primers used in real time RT-PCR

Name	Sequence
OsPR10a-RT5	5'-ATCCAGTATATCCCACCTAG-3'
OsPR10a-RT3	5'-GACCATAGAAAGGCACATAA-3'
OsPR1-RT5	5'-ATATACTACTATCGTACGCT-3'
OsPR1-RT3	5'-AGCATGCGAACTGTGTGTGT-3'
OsPR4-RT5	5'-TTGGTCATGCCTCTTTGCAT-3'
OsPR4-RT3	5'-ATTCATTATTCAGCATGATT-3'
OsPAL-RT5	5'-ACGATCGCCTGGCGAGGAGC-3'
OsPAL-RT3	5'-CTCGCCGTTCCACTCCTTGA-3'
OsLOX-RT5	5'-CGGCGTCACCGGCATGGGCA-3'
OsLOX-RT3	5'-ACATATTGGTTAAAACAGTA-3'
OsACT-RT5	5'-AGCTGTTATCGCCGTCCTCC-3'
OsACT-RT3	5'-TATTACAGTCTTTGAATAGA-3'

Gene accession number*: OsPR10a* (D38170); *OsPR1* (AF306651); *OsPR4* (AY050642); *OsPAL* (XM_015771243); *OsLOX* (NM_001068734); *OsACT* (XM_015774830)

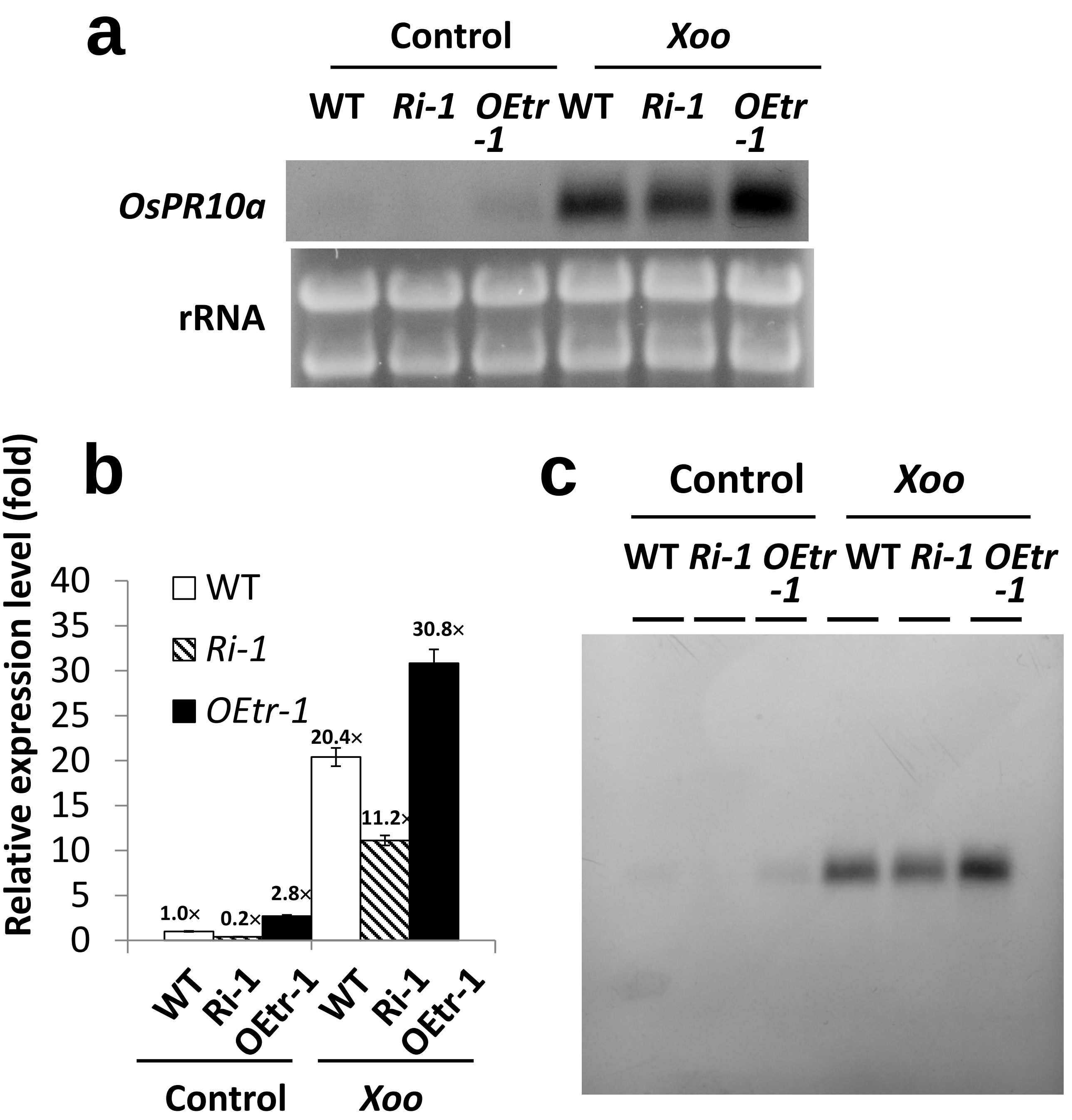

Supplementary Figure S1: (a) Northern blot analysis of *OsPR10a* expression in WT, *Ri-1* and *OEtr-1* plants. Three-week-old seedlings were treated with or without *Xanthomonas oryzae* pv. *oryzae* (*Xoo*) for 1 day. Total RNA was purified and subjected to northern blot hybridization using a probe for the *OsPR10a* coding region. rRNAs served as the quantity control. **(b)** Quantification of the northern blot hybridization signal by densitometer. The X-ray film was scanned repeatedly for three times. The relative expression level was normalized to the control of wild-type (WT) and indicated as the fold number above the error bars of individual lines. **(c)** Full image of the northern blot shown in the Figure S1a.

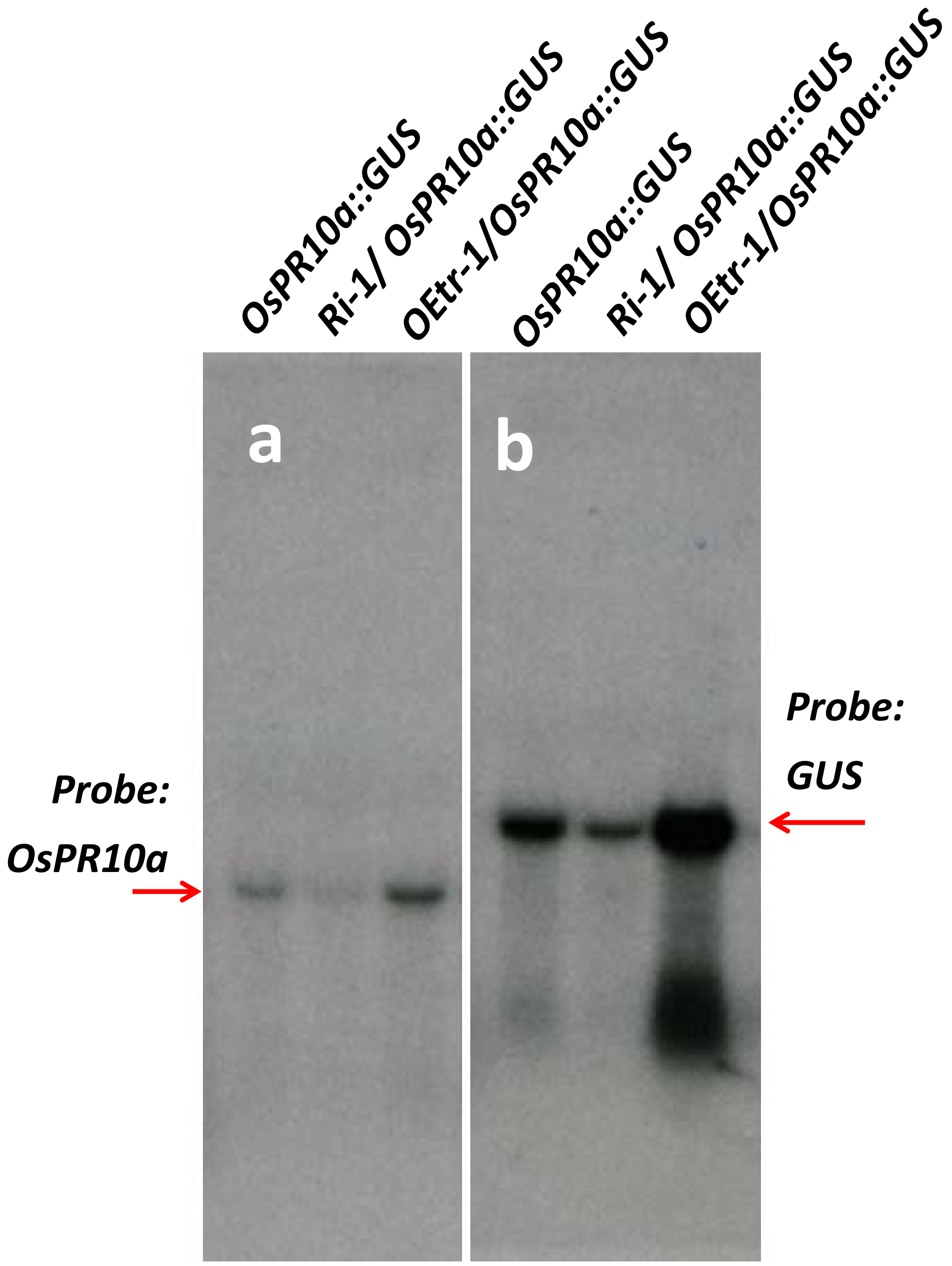

Supplementary Figure S2: Full images of the northern blot shown in the Figure 2e.

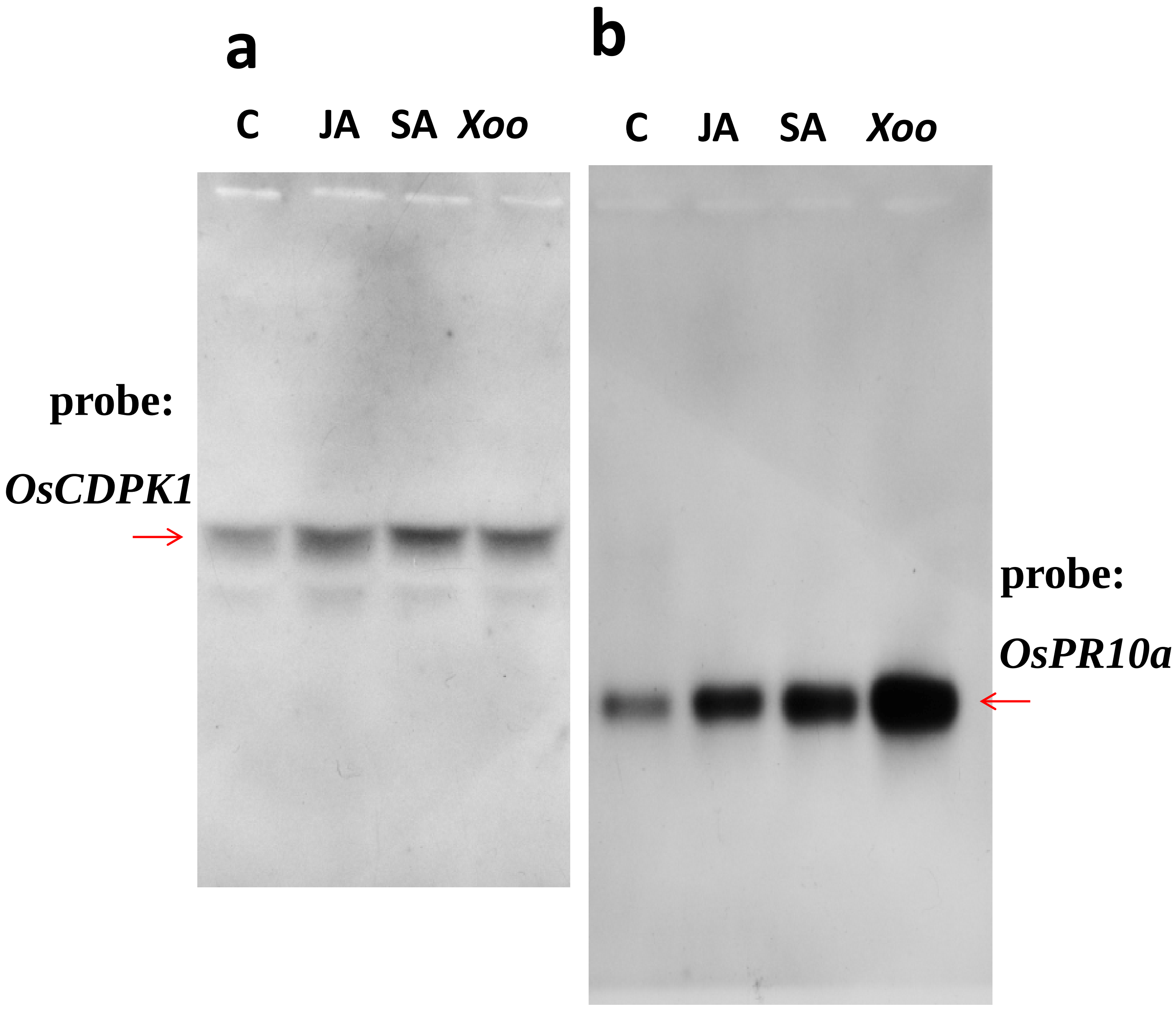

Supplementary Figure S3: Full images of the northern blot shown in the Figure 3.
Figure S3A: full image of the northern blot of Fig. 3a.
Figure S3B: full image of the northern blot of Fig. 3b.

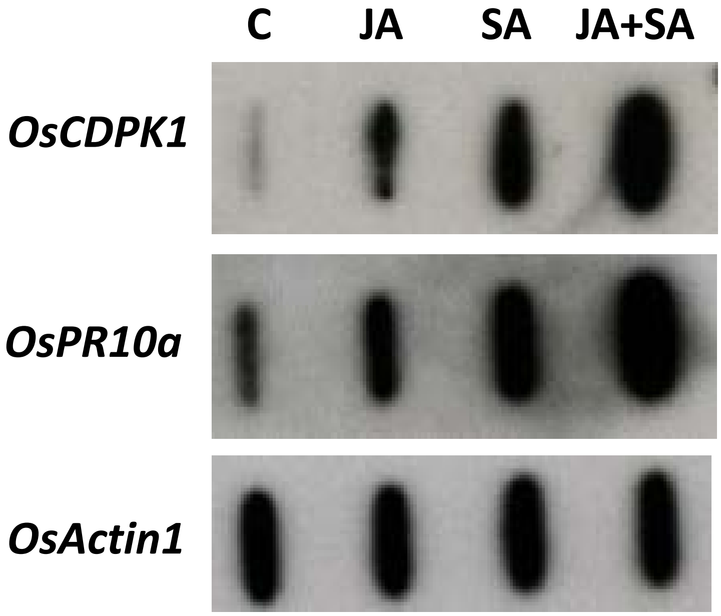

Supplementary Figure S4: Expression of *OsCDPK1* and *OsPR10a* in response to salicylic acid (SA), jasmonic acid (JA), and SA plus JA treatments.

The two-week-old seedlings were sprayed with JA (100 μM), SA (100 μM), or with JA and SA together, and grown for 1 d. Ten microgram of total RNA was purified from treated leaves and subjected to slot-blot hybridization by using the 3'-untranslated regions (3'-UTR) of *OsCDPK1* or *OsPR10a* as probes. The rice *OsActin 1* was used as quantity control. C: non-treated plants

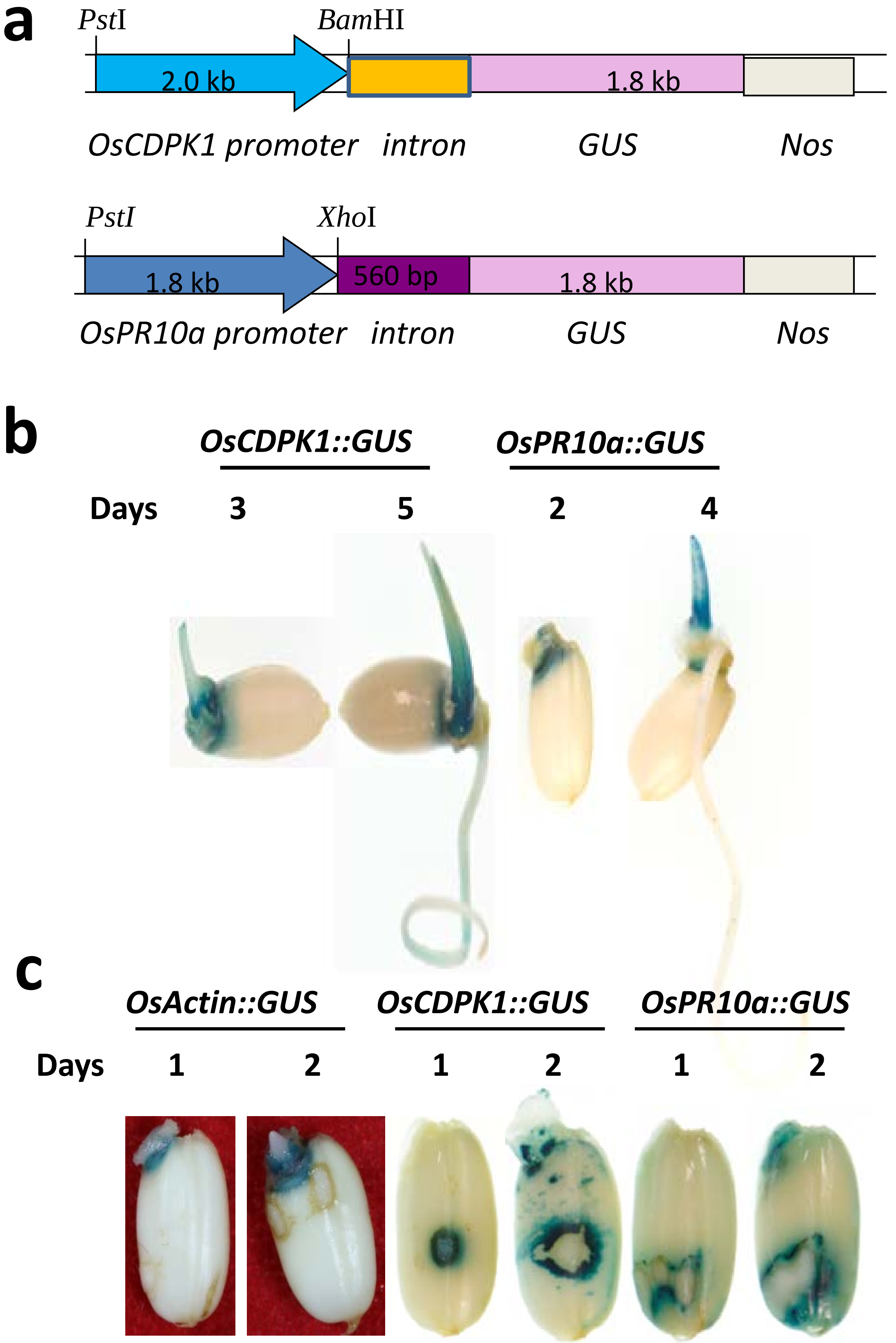

Supplementary Figure S5. Expression constructs of *OsCDPK1::GUS* and *OsPR10a::GUS* and histochemical staining of β-glucuronidase (GUS) activity in germinating seeds. **(a)** Expression constructs of *OsCDPK1::GUS* and *OsPR10a::GUS*. GUS activity in germinating seeds **(b)** without or (**c**) with pathogen infection. Seeds were germinated on ½ MS medium for 1–5 d, then stained for GUS activity

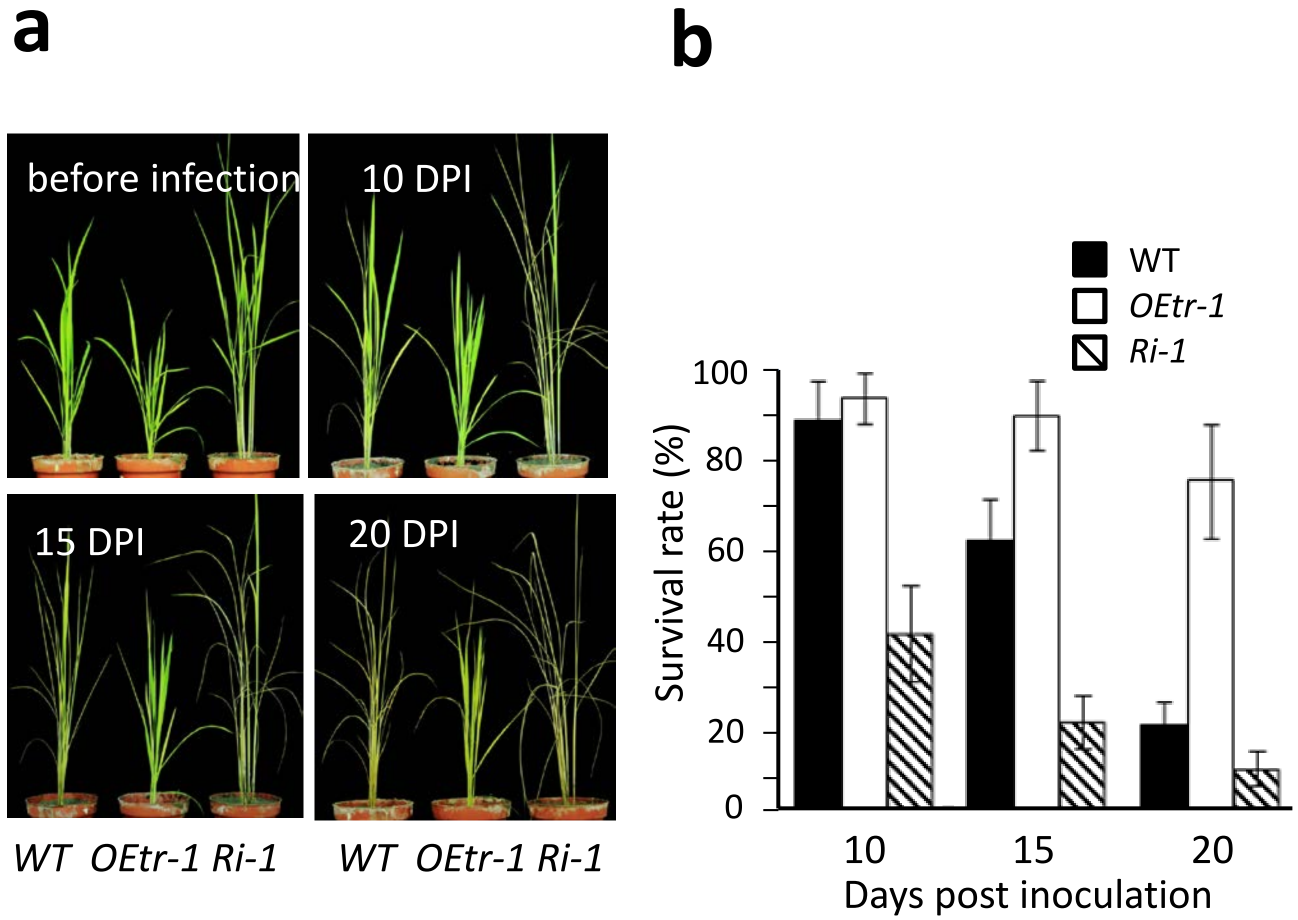

Supplementary Figure S6: Ectopic expression of constitutively active truncated form of *OsCDPK1* (*OEtr-1*) in rice showed enhanced resistance to *Xanthomonas oryzae* pv. *oryzae* (*Xoo*) infection. (**a**) The tip of all leaves on three-week-old seedlings were penetrated with a *Xoo*-contaminated needle at five different sites, then spray-inoculated with *Xoo* (1.0×10^{10} CFU/mL) once daily for 5 days. Infected plants were kept in a growth chamber (90% relative humidity; 28 $^{\circ}$C) under a 16 h light/8 h dark photoperiod for disease development. Photographs were taken at 0, 10, 15, and 20 days post-inoculation (DPI). **(b)** Quantification of plant survival after *Xoo* inoculation. The experiments were repeated three times. Different letters above bars indicate significant differences as indicated by ANOVA ($P < 0.05$). Data are shown as means $\pm$SD (n=12)

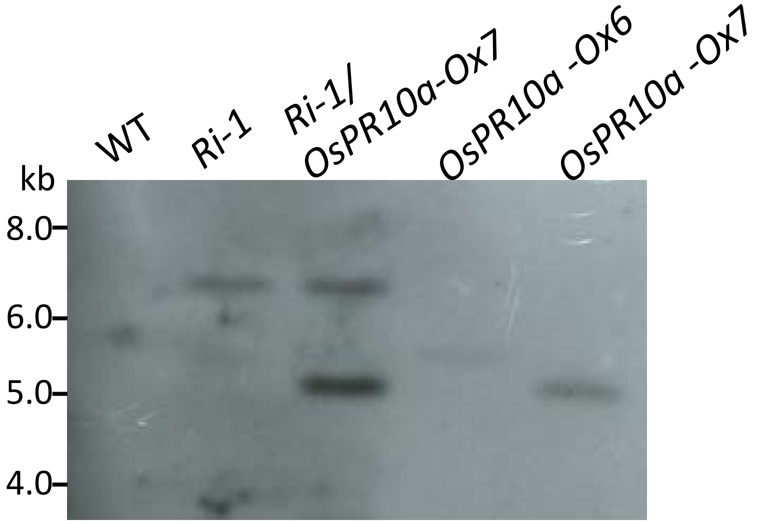

Supplementary Figure S7: Southern blot analysis of wild type (WT) and transgenic rice lines. Genomic DNA was digested with *Pst*I and subjected to Southern blot analysis using the coding region of an antibiotic resistant gene, *Hph* (*hygromycin phosphotransferase*), as the probe. The dihybrid rice line, *Ri-1*/*OsPR10a-Ox7*, harboured both transgenes

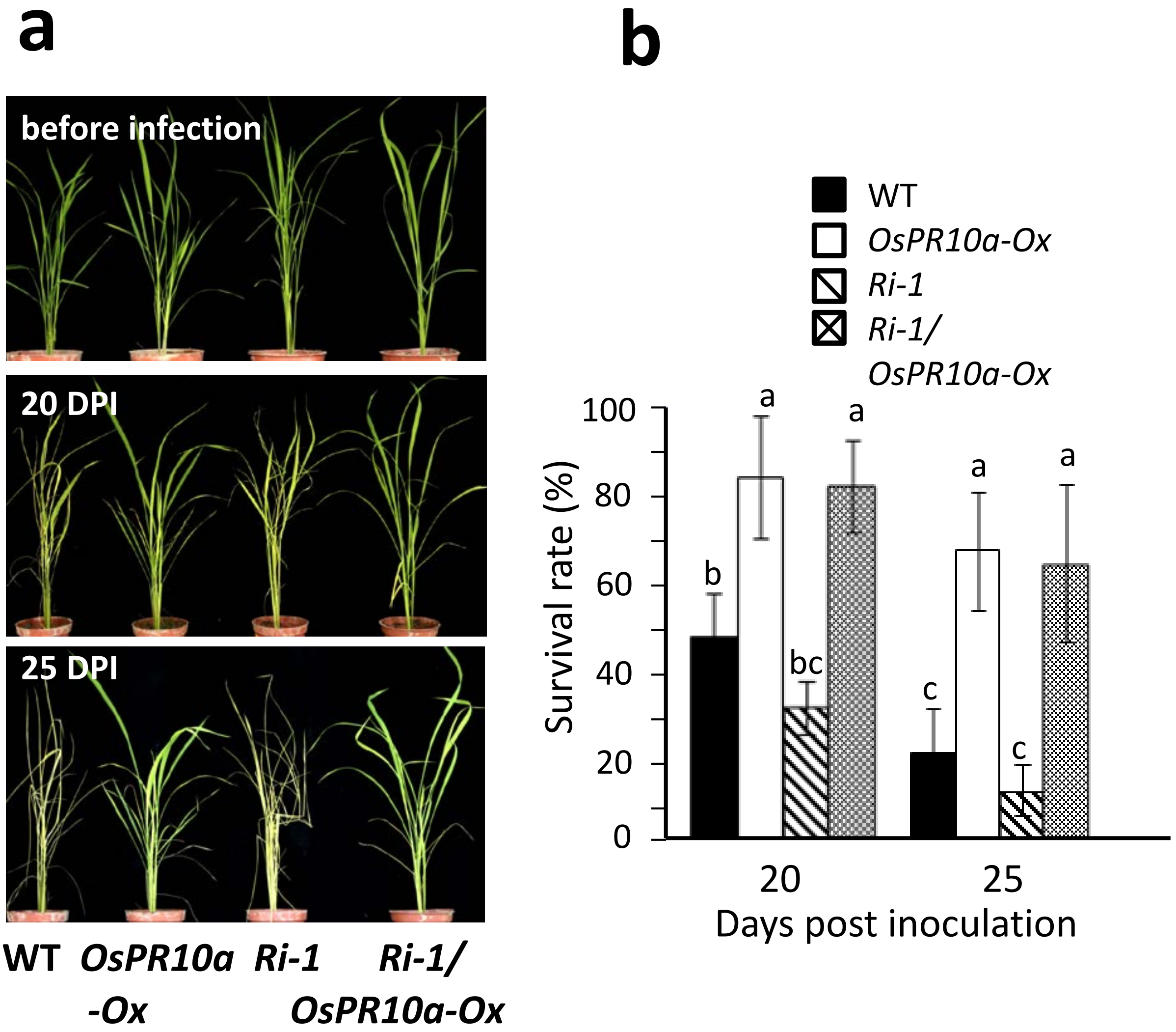

Supplementary Figure S8: Enhanced resistance to bacterial blight (*Xanthomonas oryzae* pv. *oryzae*; *Xoo*) in dihybrid transgenic rice *Ri-1/OsPR10a-Ox7*. (**a)** The leaf tip of every leaf on three-week-old seedlings was penetrated with a *Xoo*-contaminated needle at five different sites, then spray-inoculated with *Xoo* (1.0×10^{10} CFU/mL) once daily for 5 days. Infected plants were kept in a growth chamber (90% relative humidity; 28 $^{\circ}$C) under a 16 h light/8 h dark photoperiod for disease development. Photographs were taken at 0, 20, and 25 days post-inoculation (DPI). (**b)** Quantification of plant survival after *Xoo* infection. The experiments were repeated three times. Different letters above bars indicate significant differences as indicated by ANOVA ($P < 0.05$). Data are presented as means $\pm$ SD (n = 12)
